# Elevated Expression of *H19* and *Igf2* in the Female Mouse Eye

**DOI:** 10.1371/journal.pone.0056611

**Published:** 2013-02-20

**Authors:** Björn Reinius, Chandrasekhar Kanduri

**Affiliations:** Institute of Biomedicine, Department of Medical and Clinical Genetics, The Sahlgrenska Academy, University of Gothenburg, Gothenburg, Sweden; Florida State University, United States of America

## Abstract

The catalogue of genes expressed at different levels in the two sexes is growing, and the mechanisms underlying sex differences in regulation of the mammalian transcriptomes are being explored. Here we report that the expression of the imprinted non-protein-coding maternally expressed gene *H19* was female-biased specifically in the female mouse eye (1.9-fold, p = 3.0E−6) while not being sex-biased in other somatic tissues. The female-to-male expression fold-change of *H19* fell in the range expected from an effect of biallelic versus monoallelic expression. Recently, the possibility of sex-specific parent-of-origin allelic expression has been debated. This led us to hypothesize that *H19* might express biallelically in the female mouse eye, thus escape its silencing imprint on the paternal allele specifically in this tissue. We therefore performed a sex-specific imprinting assay of *H19* in female and male eye derived from a cross between *Mus musculus* and *Mus spretus*. However, this analysis demonstrated that *H19* was exclusively expressed from the maternal gene copy, disproving the escape hypothesis. Instead, this supports that the female-biased expression of *H19* is the result of upregulation of the single maternal. Furthermore, if *H19* would have been expressed from both gene copies in the female eye, an associated downregulation of Insulin-like growth factor 2 (*Igf2*) was expected, since *H19* and *Igf2* compete for a common enhancer element located in the *H19/Igf2* imprinted domain. On the contrary we found that also *Igf2* was significantly upregulated in its expression in the female eye (1.2-fold, p = 6.1E−3), in further agreement with the conclusion that *H19* is monoallelically elevated in females. The female-biased expression of *H19* and *Igf2* specifically in the eye may contribute to our understanding of sex differences in normal as well as abnormal eye physiology and processes.

## Background

**Figure 1:**
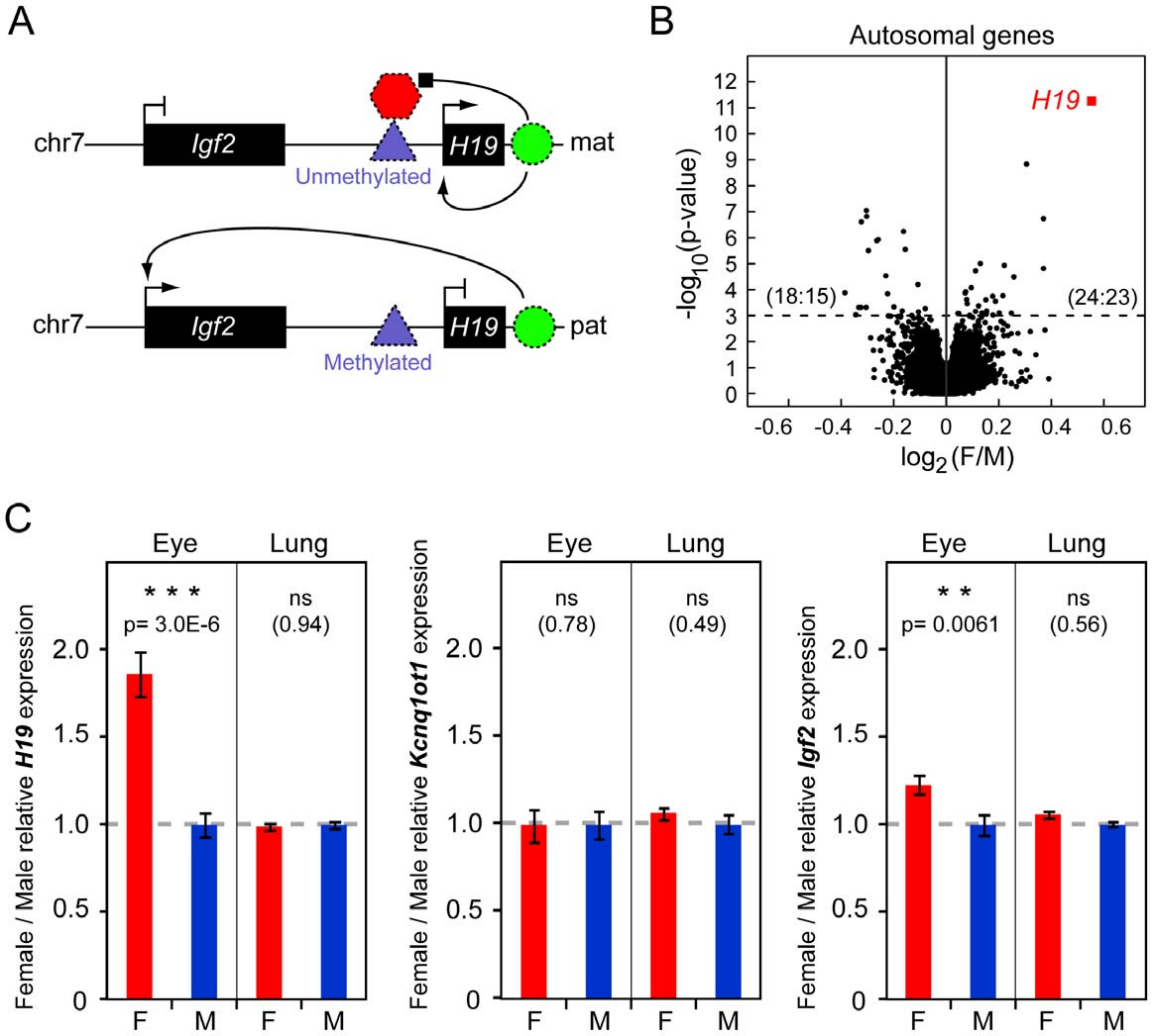
Gene expression analysis. A. Schematic model of allelic regulation in the *H19/Igf2* imprinted domain, and premise for the experimental approach. The imprinting control region (ICR, triangle) is unmethylated on the maternal allele (*mat*), allowing for the expression of *H19_mat_* and the binding of the CCCTC-binding factor (hexagon) which insulates *Igf2_mat_* from interaction with an enhancer element (circle) located downstream of *H19*. Thus *Igf2_mat_* is normally silenced when *H19_mat_* is expressed. In contrast, on the paternal allele (*pat*), the ICR is methylated, preventing expression of *H19_pat_* and blocking CCCTC-binding to the ICR which allows *Igf2_pat_* to interact with the enhancer element and to be expressed. **B.** Volcano plot, separating female-biased (upper right quadrant) and male-biased (upper left quadrant) autosomal genes in the mouse eye in our microarray screen. *H19* (red square) is identified as a candidate female-biased gene (p = 1.3E−12, female/male fold-change = 1.5). y-axis: –log_10_(p-value, two-sided t-test), x-axis: log_2_(female/male) expression ratio. The dotted line represents the significance threshold p = 0.001, and numbers within parenthesis denote the number of significant probes : unique genes in each sex. n_females_ = 88, n_males_ = 88. **C.** RT-qPCR assays of female (F) and male (M) eye and lung tissues. Expression is normalized to the geometric mean of *Gapdh* and *Actb* and shown relative to the mean male expression in each tissue. P-values are given according to a two-sided t-test and error bars denote standard error of the mean. n_females, eye_ = 19, n_males, eye_ = 19, n_females, lung_ = 16, n_males, lung_ = 18.

Mounting evidence has cemented our awareness of the importance of taking molecular sexual dimorphism into account for reaching a fuller understanding of normal physiology [1], [2], [3], [4], [5] as well as pathological conditions with sex-biased characteristics [6], [7], [8]. Sex differences in autosomal gene expression can vary extensively between tissues [9], [10] and are known to be regulated not only by sex hormones, but also directly by genes located on the X and Y chromosomes [11], and even via interaction with the X-inactive chromatin in female cells [12]. Recently a new mode of sexual gene expression bias was reported in the mouse. An RNA-sequencing analysis showed that sex-specific effects on the expression of the paternal versus the maternal allele (i.e. sex-specific imprinting effects) were wide-spread [13], although the validity of some of these results has later been questioned [14]. Moreover, a quantitative trait locus analysis reported sex-dependent imprinting effects on complex traits in mice [15]. However, the existence of sex-specific imprinting effects is not yet widely accepted, and possible underlying mechanisms remain to be described. Imprinted genes are of particular interest for understanding the evolution of sexual dimorphism, particularly in the context of competition between the sexes. The parental conflict hypothesis [16] states that the skew in maternal versus paternal optimal investment during the generation of offspring has led to the evolution of imprinted genes. The father’s fitness can be increased by delivering genes to the offspring that promote increased energy consumption at the expense of the mother, while the mother responds by activating growth inhibiting genes. The first imprinted locus to be identified in mouse, and likely the most studied imprinted locus, is the *H19*/*Igf2* domain [17], [18], [19]. This locus encodes the paternally expressed Insulin growth factor 2 (*Igf2*), which is a major fetal growth factor, thus fitting well within the parental conflict hypothesis. The other gene in this locus, *H19*, is a long non-coding RNA expressed from the maternal allele. The specific expression of paternal *Igf2* and maternal *H19* is achieved by a mutually exclusive interaction with an enhancer element located in the *H19*/*Igf2* imprinting domain (**Figure 1A**) [20], [21], [22].

Recently, we performed a large-scale microarray analysis of sexually dimorphic gene expression in somatic mouse tissues incorporating more than 700 microarray hybridizations [23]. In this analysis, *H19* was identified as the top female-biased gene candidate among expressed autosomal transcripts in the mouse eye (1.5 fold, p = 1.3E−12, **Figure 1B**), while *H19* was not significantly sex-biased in other tissues analyzed (lung, liver, kidney, striatum, and hippocampus). *H19* is expressed in several compartments of the mouse eye, including the retina, iris, ciliary bodies, eyecup and the cornea (**Figure S1**). Given that the female-bias of *H19* fell in the range expected by an effect of biallelic versus monoallelic expression [10], [24], and the recent reports of a large number of imprinted genes showing sex-specific imprinting effects [13], we here investigated the possibility that *H19* might “escape” its silencing imprint on the paternal allele specifically in the female eye. If so, this would open new avenues for the exploration of the mechanisms behind *H19/Igf2* imprinting, since the molecular characteristics of different epigenetic states of the *H19/Igf2* imprinted domain could be compared both within a tissue (by comparing male and female eye) and between tissues (by comparing female eye with other tissues).

## Results and Discussion

### Gene Expression Analysis

To validate the eye-specific female-bias of *H19* expression we performed an RT-qPCR analysis of female and male eye, and included lung as a control tissue, using samples that were biologically independent from the specimens employed in the microarray experiments. As expected, this analysis confirmed a female-elevated expression of *H19* in the eye (1.9 fold, p = 3.0E−6, **Figure 1C**) while no difference was observed in the lung. To ensure that the sex-bias was specific to *H19* transcription, and not a general effect in the eye tissues, we analyzed the expression of *Kcnq1ot1*, an imprinted long non-coding RNA located at mouse chromosome 7, 0.5 Mbp downstream of *H19*, and found no sex difference in expression of this control gene (**Figure 1C**).

### Sex-specific Imprinting Assay of H19

**Figure 2:**
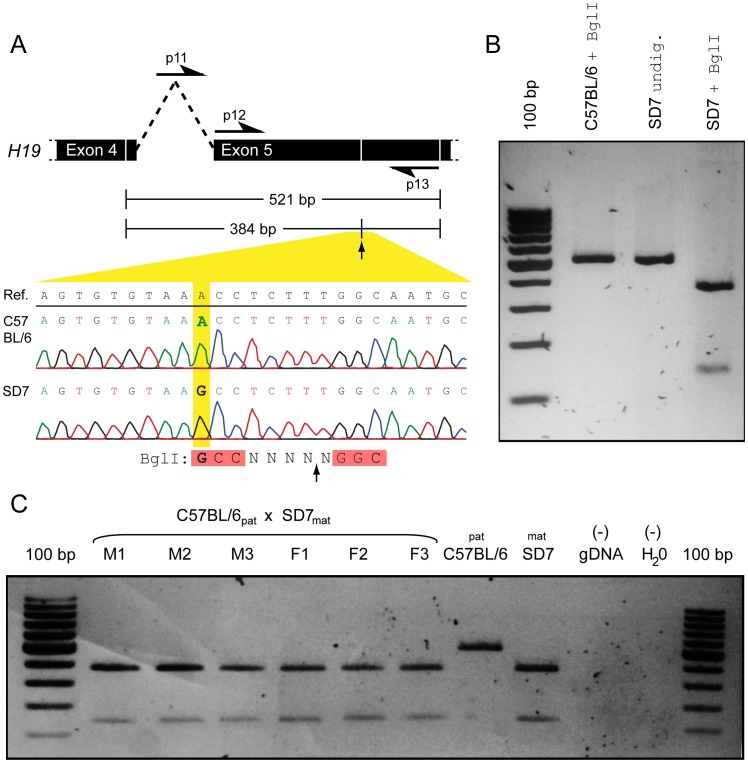
Sex-specific imprinting assay of *H19*. A. RFLP experimental design. DNA sequencing of C57BL/6 and SD7 confirmed an SD7-specific BglI restriction site located within *H19* exon 5. p11, p12 and p13 designate the locations of the RFLP primers listed in Table 1. **B.** Confirmation of SD7-specificity of the BglI restriction. PCR products of *H19* amplified from BL6 and SD7 gDNA (using primers p12 and p13) digested with BglI, and an undigested SD7 sample. **C.** Imprinting assay of *H19* in male and female mice. PCR products of *H19* amplified from eye cDNA derived from three F1 males (M1-3) and three F1 females (F1-3) of the ♂C57BL/6×♀SD7 cross (using primers p11 and p13) digested with BglI. Controls for the paternal (C57BL/6) and maternal (SD7) allele, and negative controls for the PCR are shown to the right.

Next, to investigate whether the upregulation of *H19* in the female eye was an effect of expression from both alleles (i.e. loss of imprinting) or an effect of upregulation of the maternal gene copy, we conducted a sex-specific imprinting assay. To distinguish between the paternal and maternal allele, we generated F1 offspring carrying polymorphisms within the *H19* gene by crossing the mouse strains C57BL/6 (♂) and SD7 (♀). SD7 is a mouse strain maintained on a C57BL/6 background with the distal arm of chromosome 7 derived from the distant mouse subspecies *Mus spretus* [25]. Thus the F1s were heterozygous for the *H19* allele. To identify single nucleotide polymorphisms (SNPs), we amplified and partially sequenced *H19* in SD7 and C57BL/6. We identified one SNP in exon 4; five SNPs in exon 5; and 2 intronic polymorphisms (**Figure S2**. The SD7 sequence was deposited in GenBank: accession number JX869491). This, together with a Restriction Fragment Length Polymorphism (RFLP) analysis, confirmed the existence of an SD7-specific BglI restriction site within exon 5 (**Figure 2A and B**). We then carried out an RFLP analysis of *H19* cDNA derived from the eye of three male and three female F1s. This analysis clearly showed that *H19* was exclusively expressed from the maternal allele in males as well as in females (**Figure 2C**), disproving the hypothesis that *H19* escape imprinting in the female eye. From this it follows that the female-elevated expression is derived from an eye-specific upregulation of maternal *H19* expression relative to that in males. Furthermore, if *H19* would have been biallelically expressed in the female eye we expected an associated downregulation of *Igf2* (See the model in **Figure 1A**), and we therefore performed an RT-qPCR analysis of *Igf2*. On the contrary, and in further refutation to the escape hypothesis, we found that also *Igf2* was significantly female-biased in the mouse eye (1.2 fold, p = 0.0061, **Figure 1C**). These results conclude that female-specific elevated expression of *H19* is not due to loss of imprinting, rather due to hitherto unknown female eye-specific transcriptional regulatory mechanisms.

In the context of our results, it is of interest that epigenetic sex differences in the imprinted *H19*/*Igf2* domain have been previously observed. For example, an analysis of embryonic germ cells showed that *H19* is more strongly methylated in males than in females at 11.5 and 12.5 dpc, and likewise for *Igf2* at 12.5 dpc [26]. This is an intrinsic cell-autonomous effect of the sex chromosome complement (XY versus XX) and not an effect of differences in systemic sex hormone levels [27]. These results correlate with our observation of robust female-biased expression of *H19* and slight female-bias of *Igf2*. Sex differences in a range of aspects in eye anatomy and function are known in normal as well as in disease conditions (reviewed in [28]). For example, malignant glaucoma is about twice as common in females as in males [29]. Cataloguing sex-biased molecular characteristics will be crucial for approaching a more complete understanding sex differences in eye physiology. Of further interest is that *H19* and *Igf2* are both oncogenes known to be deregulated in various types of tumors [30], [31], [32], [33], [34], [35], *H19* often being overexpressed in the tumorous tissues. The functional consequences and the evolutionary ground of the eye-specific female-elevated expression of *H19* and *Igf2* are now open for further in-depth investigation.

### Conclusion

We showed that the expression of the imprinted genes *H19* and *Igf2* is female-biased in the mouse eye, while not sex-biased in other tissues analyzed. We speculated that the elevation of *H19* might be a result of escape from its silencing genomic imprint on the paternal allele specifically in the female eye. Our imprinting assay showed that this was not the case, and therefore *H19* upregulation emerges from the single maternal allele from which it is expressed. The awareness of sex differences in gene expression is important for understanding the molecular basis of sexual dimorphism in normal physiology and in disease, as well as for understanding of the evolution of imprinted and non imprinted traits with sex-biased characteristics.

## Materials and Methods

### Ethics Statement

The use of the experimental animals in this study was approved by the appropriate Swedish ethical committee, permits: c79/9 and 307-2011, Jordbruksverket.

### Gene Expression Analysis

#### Microarray analysis

The eye microarray analysis included 176 RMA normalized microarrays (n_female_ = 88, n_male_ = 88, MOE340 Affymetrix, *Mus musculus* BXD strains) and is described in detail elsewhere [23]. This data is available in the GeneNetwork depository: http://www.genenetwork.org, “Hamilton Eye Institute Mouse Eye M430v2 Data Set (Sept08) RMA”, Accession number: GN207. The significance criterion was p<0.001, two-sided t-test. We confirmed that the female-bias of *H19* was not unique to BXD strains by similar analysis of a panel of female/male pairs from 20 different mouse subspecies and strains (p = 0.0006 two-sided paired t-test, mean fold-change: 1.2, n_female_ = 20, n_male_ = 20, **Table S1**). Expression data for **Figure S1** was obtained from BioGPS [36] (http://biogps.org, GeneAtlas MOE430 gcrma dataset; 1448194_a_at). *RT-qPCR analysis:* Total RNA was extracted from male and female eyes (n_female_ = 19, n_male_ = 19, *Mus musculus* C57BL/6 strain) and lung (n_female_ = 16, n_male_ = 18) after weaning (>P21, Swedish ethical committee permit: c79/9, Jordbruksverket) using Trizol (Invitrogen). RNA was reverse transcribed using a Dynamo cDNA synthesis kit F-470L (Finnzymes) and the following reagents: 0.95 µg total RNA, 15 ng/µl random hexamers, 10 U M-MuLV RNase H- reverse transcriptase, 1 × RT buffer, ddH_2_O, in a total reaction volume of 20 µl. Incubations were performed in a PTC-100 Peltier Thermal Cycler (MJ Research): 25°C; 10 min, 37°C; 45 min, 85°C; 5 min. cDNA samples were subsequently diluted 1∶10 in ddH_2_O. Reactions contained 0.3 µM of each primer (**Table 1A**), 1×Power SYBR Green Master Mix (Applied Biosystems), 4 µl diluted cDNA sample and ddH_2_O in a total reaction volume of 30 µl. Thermal cycles were: 50°C; 2 min, 95°C; 10 min, 40 cycles: 95°C; 15 s, 60°C; 1 min. To ensure that single PCR products of intended lengths were amplified, a melting program was executed subsequent to the quantifications and bands of expected sizes were inspected after gel electrophoresis. Copy numbers were determined relative to a cDNA dilution series. Expression was normalized to the geometric mean of *Gapdh* and *Actb*. The criterion for differential expression was p<0.05, two-tailed t-test. Information on overall expression of *H19* and *Igf2* in eye versus lung is included in **Figure S3**.

**Table 1:** Primers.

Table 1a. qPCR primers.		
*H19*	forward	AGAGGACAGAAGGGCAGTCA
	reverse	TGGGTGGACAATTAGGTGGT
*Kcnq1ot1*	forward	AGAGGACAGAAGGGCAGTCA
	reverse	TGGGTGGACAATTAGGTGGT
*Igf2*	forward	CGCTTCAGTTTGTCTGTTCG
	reverse	AAGCAGCACTCTTCCACGAT
*Gapdh*	forward	GCCTTCCGTGTTCCTACC
	reverse	GCCTGCTTCACCACCTTC
*Actb*	forward	TGTTACCAACTGGGACGACA
	reverse	GGGGTGTTGAAGGTCTCAAA
**Table 1b** **. ** ***H19*** ** sequencing primers.**		
*H19* seq	forward	CCAGGTCTCCAGCAGAGGT
	reverse	TTTATTGATGGACCCAGGAC
**Table 1c** **. ** ***H19*** ** RFLP primers.**		
*H19* p11	forward	TGCTCCAAGGTGAAGCTGAAAG
*H19* p12	forward	GTGAAGCTGAAAGAACAGATGGTG
*H19* p13	reverse	GTAGGGCATGTTGAACACTTTATG

The primer sequences are given 5′ to 3′.

### Imprinting Assay

#### DNA sequencing

PCR amplification of *H19* gDNA for Sanger sequencing was performed: 94°C; 2 min, 35× (94°C; 10 s, 55°C; 40 s, 72°C; 90 s), 72°C; 10 min. Each reaction contained 100 ng gDNA, 0.75 U DreamTac DNA polymerase (Fermentas), 2.5 µl DreamTaq buffer including MgCl_2_ (Fermentas), 0.3 µM of each primer (**Table 1B**), 0.4 µM dNTPs, ddH_2_O to a total volume of 25 µl. DNA sequencing was performed at GATC-Biotech, Stockholm, using the primers in **Table 1B**. *RFLP analysis:* Eye and lung tissues were dissected from F1 mice (♂C57BL/6×♀SD7) after weaning. Total RNA was extracted using Trizol (Invitrogen) and treated with DNase I (Fermentas). Reverse transcription and PCR was performed as described above using the primers listed in **Table 1C**. 10 µl PCR product was incubated with 20 U BglI restriction enzyme (Fermentas), 3 µl Buffer O (Fermentas), ddH_2_O to a total volume of 30 µl, 37°C; 28 h. Fragments were separated on a 2% agarose gel and stained with ethidium bromide. This protocol was based on an imprinting assay described by Sasaki et al. [37]. The Swedish ethical committee permit for the C57BL/6 and SD7 animals used in the imprinting assay is 307-2011, Jordbruksverket.

## Supporting Information

Figure S1 **The expression level of** ***H19*** **in a panel of mouse tissues, including sub compartments of the eye (1448194_a_at, MOE430 Affymetrix, BioGPS:** http://biogps.org **).**
(PDF)

Figure S2 **A.** Chromatogram showing eight C57BL/6:SD7 polymorphisms (pm) within the *H19* gene, as identified by Sanger sequencing. **B.** Alignment between a *H19* mouse reference sequence and the SD7 sequence.
(PDF)

Figure S3 **The overall expression level of** ***H19*** **(n_females_ = 19, n_males_ = 19) and** ***Igf2*** **(n_females, lung_ = 16, n_males, lung_ = 16) in eye and lung.** P-values are given according to a two-sided t-test and error bars denote standard error of the mean.
(PDF)

Table S1 Eye *H19* intensities from analysis of 1 female and 1 male microarray hybridization in 20 mouse strains/subspecies (1448194_a_at, MOE340 Affymetrix, GeneNetwork depository: http://www.genenetwork.org, Accession number: GN207). RNA pools of 4–8 eyes from 2–4 individuals are included in each hybridization.
(PDF)
