# Supplementary figures and images for: Elevated Expression of H19 and Igf2 in the Female Mouse Eye
Source: PLoS One. 2013 Feb 20;8(2):e56611. doi: 10.1371/journal.pone.0056611 (PMC3577879; doi:10.1371/journal.pone.0056611)

Figure S1

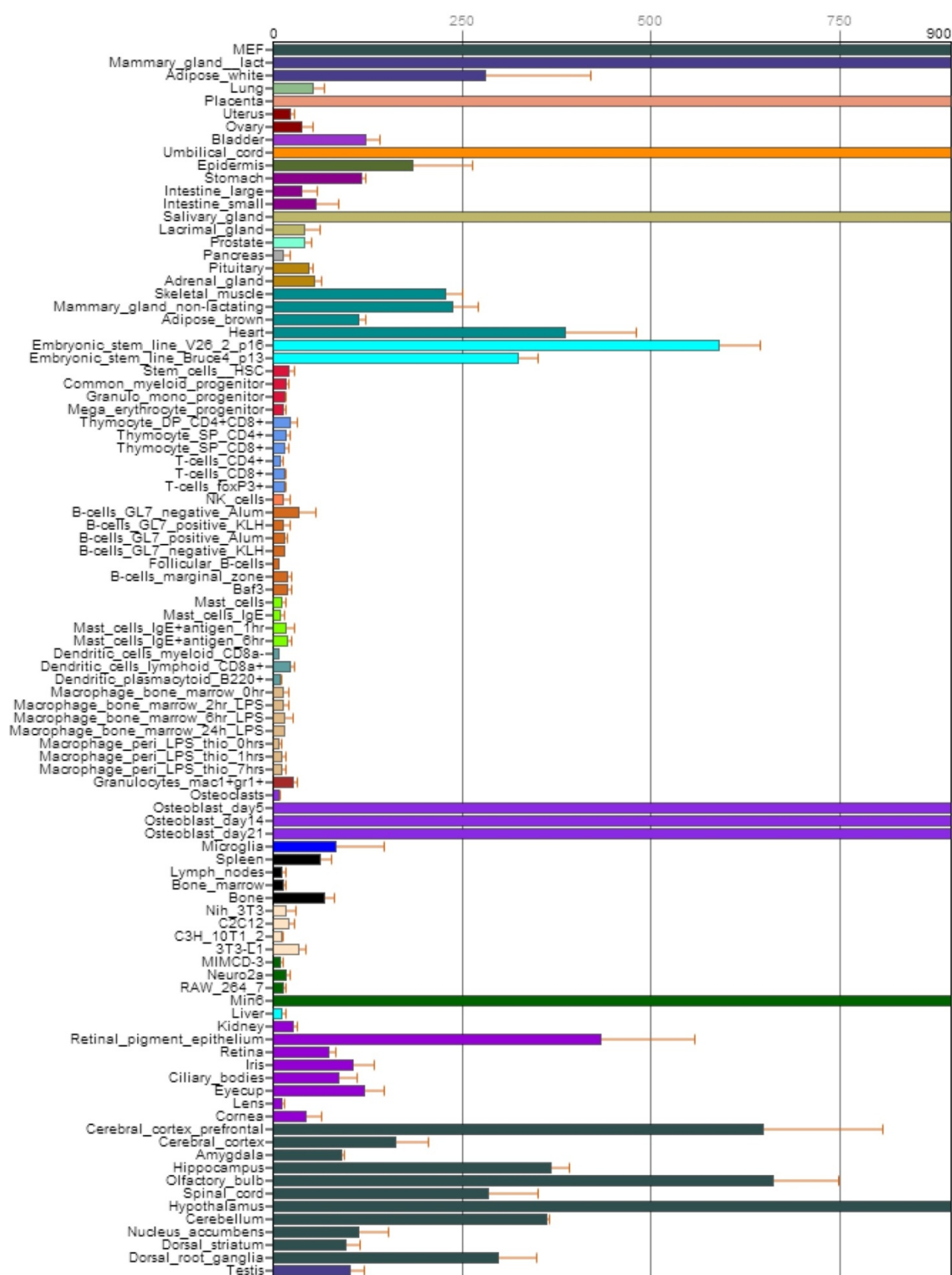

Supplement: Figure S1 — The expression level of H19 in a panel of mouse tissues, including sub compartments of the eye (1448194_a_at, MOE430 Affymetrix, BioGPS: http://biogps.org ). (PDF) [file pone.0056611.s001.pdf]

Figure S3

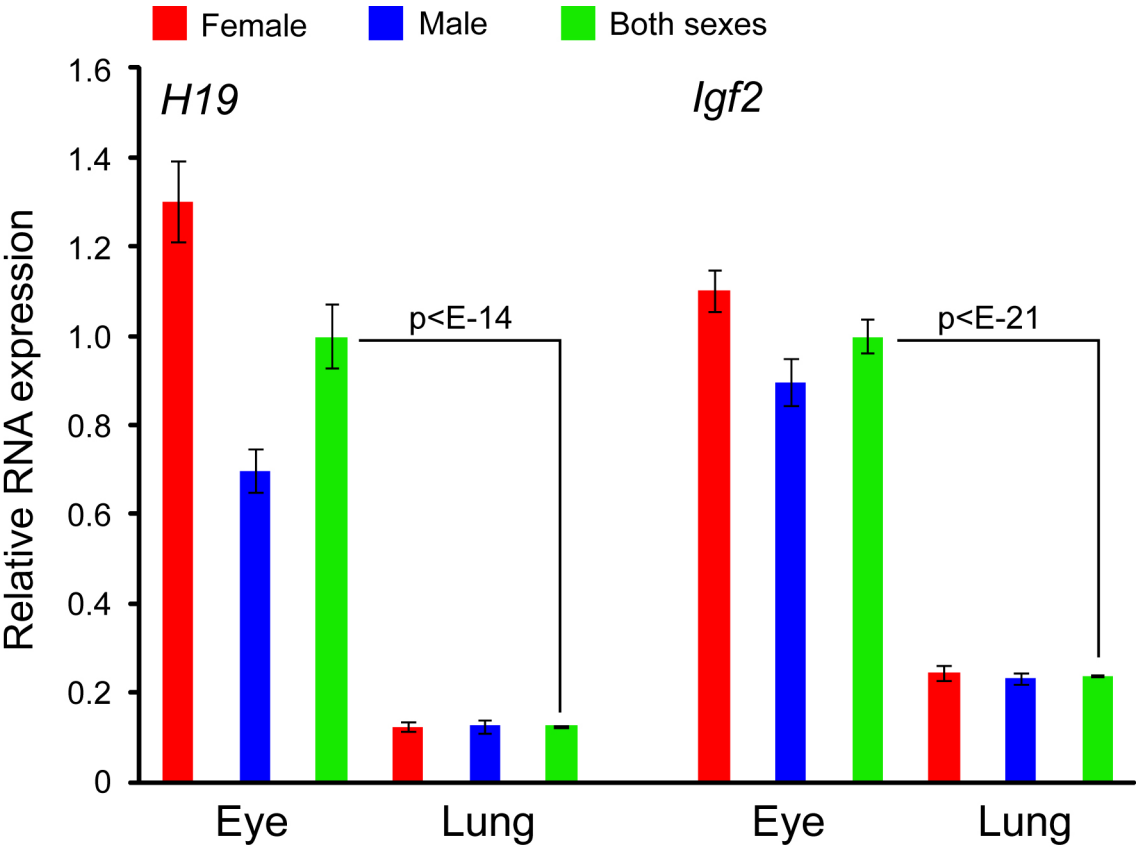

Supplement: Figure S3 — The overall expression level of H19 (nfemales = 19, nmales = 19) and Igf2 (nfemales, lung = 16, nmales, lung = 16) in eye and lung. P-values are given according to a two-sided t-test and error bars denote standard error of the mean. (PDF) [file pone.0056611.s003.pdf]
