# Supplementary material for: Elevated Expression of H19 and Igf2 in the Female Mouse Eye
Source: PLoS One. 2013 Feb 20;8(2):e56611. doi: 10.1371/journal.pone.0056611 (PMC3577879; doi:10.1371/journal.pone.0056611)
Supplement: Figure S2 — A. Chromatogram showing eight C57BL/6:SD7 polymorphisms (pm) within the H19 gene, as identified by Sanger sequencing. B. Alignment between a H19 mouse reference sequence and the SD7 sequence. (PDF) [file pone.0056611.s002.pdf]

Figure S2 A

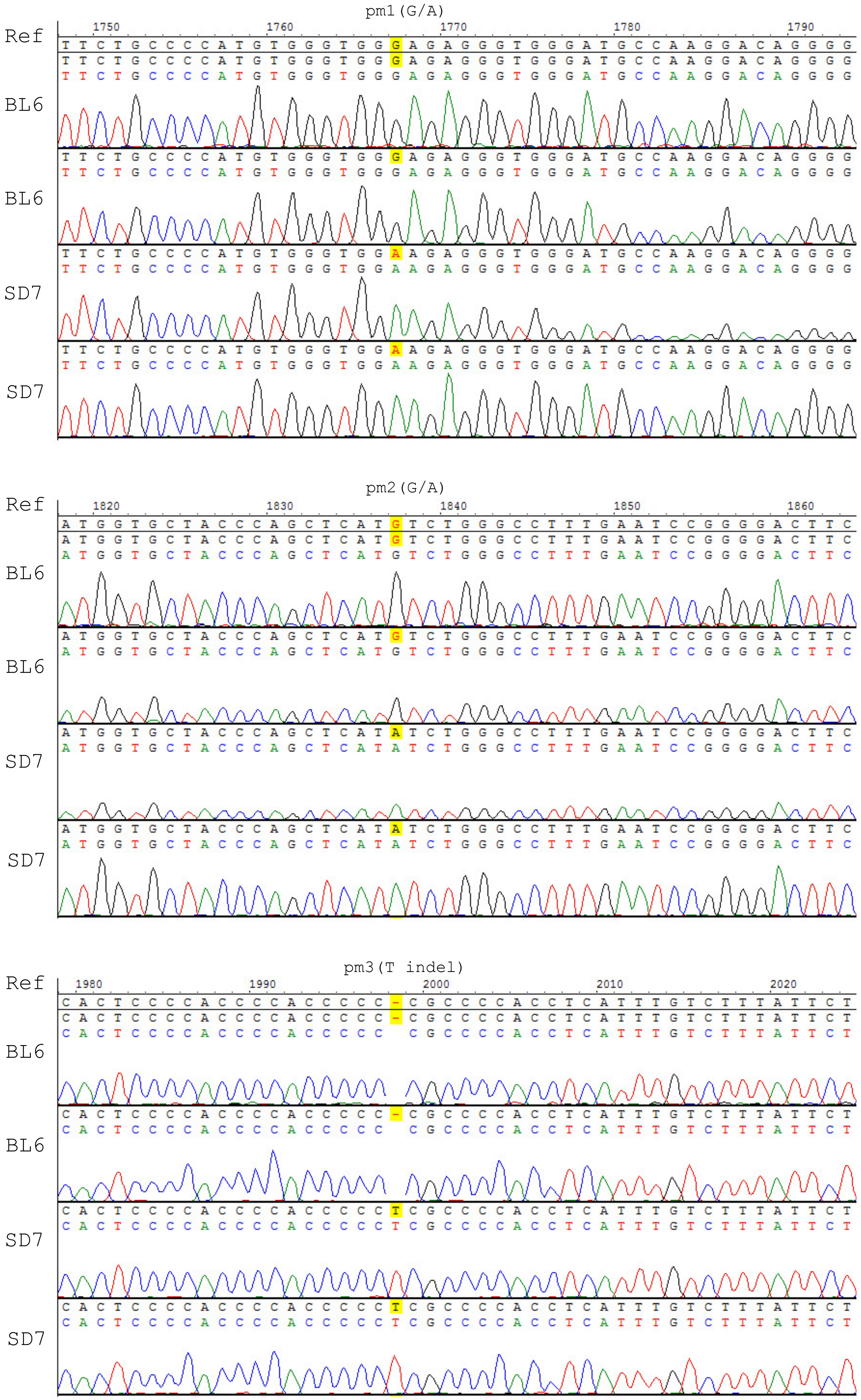
pm1 (G/A)
Ref
1750
1760
1770
1780
1790
TTCTGCCCCATGTGGGTGGGAGAGGGTGGGATGCCAAGGACAGGGG
BL6
TTCTGCCCCATGTGGGTGGGAGAGGGTGGGATGCCAAGGACAGGGG
BL6
TTCTGCCCCATGTGGGTGGGAGAGGGTGGGATGCCAAGGACAGGGG
SD7
TTCTGCCCCATGTGGGTGGAAGAGGGTGGGATGCCAAGGACAGGGG
SD7
TTCTGCCCCATGTGGGTGGAAGAGGGTGGGATGCCAAGGACAGGGG
pm2 (G/A)
Ref
1820
1830
1840
1850
1860
ATGGTGCTACCCAGCTCATGTCTGGGCCTTTGAATCCGGGGACTTC
BL6
ATGGTGCTACCCAGCTCATGTCTGGGCCTTTGAATCCGGGGACTTC
BL6
ATGGTGCTACCCAGCTCATGTCTGGGCCTTTGAATCCGGGGACTTC
SD7
ATGGTGCTACCCAGCTCATATCTGGGCCTTTGAATCCGGGGACTTC
SD7
ATGGTGCTACCCAGCTCATATCTGGGCCTTTGAATCCGGGGACTTC
pm3 (T indel)
Ref
1980
1990
2000
2010
2020
CACTCCCCACCCCACCCCC-CGCCCCACCTCATTTGTCTTTATTCT
BL6
CACTCCCCACCCCACCCCC-CGCCCCACCTCATTTGTCTTTATTCT
BL6
CACTCCCCACCCCACCCCC-CGCCCCACCTCATTTGTCTTTATTCT
SD7
CACTCCCCACCCCACCCCCTCGCCCCACCTCATTTGTCTTTATTCT
SD7
CACTCCCCACCCCACCCCCTCGCCCCACCTCATTTGTCTTTATTCT

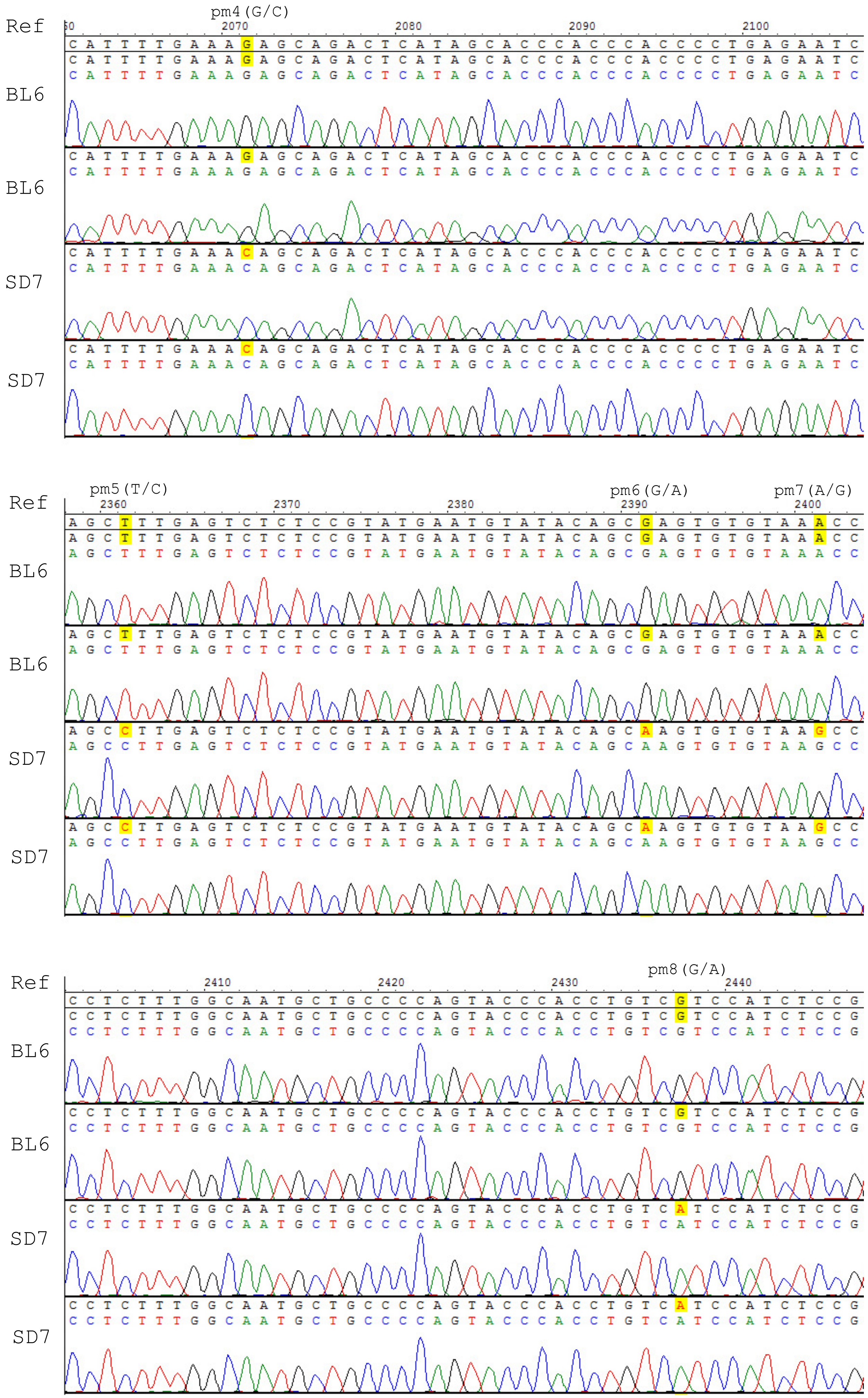
pm4 (G/C)
Ref
2070
2080
2090
2100
CATTTTGAAAGAGCAGACTCATAGCACCCACCCACCCCTGAGAATC
BL6
BL6
SD7
SD7
pm5 (T/C)
pm6 (G/A)
pm7 (A/G)
Ref
2360
2370
2380
2390
2400
AGCTTTGAGTCTCTCCGTATGAATGTATACAGCGAGTGTGTAAACC
BL6
BL6
SD7
SD7
pm8 (G/A)
Ref
2410
2420
2430
2440
CCTCTTTGGCAATGCTGCCCCAGTACCCACCTGTCGTCCATCTCCG
BL6
BL6
SD7
SD7

Figure S2 B

```
Thu Apr 26, 2012 18:59 +0200
H19_Ref_ENSMUST00000136359_NCBIM37_chr7_149761434-149764048_str_-1.str from 1 to 2615
Alignment to
H19_SD7_Seq.ape from 1 to 915

Matches(|):907
Mismatches(#):7
Gaps( ):1702
Unattempted(.):0
      Exon1
               *         *         *         *         *         *         *         *         *         *
   1 gggtgtgggagggggggtgggggggtgggggtgggggggtatcggggaaactggggaagatgggagagctggaggagagtcgtggggtccgaggagcacctcg 100

   1 ~~~~~~~~~~~~~~~~~~~~~~~~~~~~~~~~~~~~~~~~~~~~~~~~~~~~~~~~~~~~~~~~~~~~~~~~~~~~~~~~~~~~~~~~~~~~~~~~~~~~ 1

               *         *         *         *         *         *         *         *         *         *
 101 gcatctggagtctggcaggaatgttgaaggactgaggggctagctcaggcagagcaaaggcatcgcaaaggctggaaaacatcggagtgaagctgaaggg 200

   1 ~~~~~~~~~~~~~~~~~~~~~~~~~~~~~~~~~~~~~~~~~~~~~~~~~~~~~~~~~~~~~~~~~~~~~~~~~~~~~~~~~~~~~~~~~~~~~~~~~~~~ 1

               *         *         *         *         *         *         *         *         *         *
 201 cctgagctagggttggagaggaatggggagccagacattcatcccggttacttttggttacaggacgtggcggctggtcggataaaggggagctgctggg 300

   1 ~~~~~~~~~~~~~~~~~~~~~~~~~~~~~~~~~~~~~~~~~~~~~~~~~~~~~~~~~~~~~~~~~~~~~~~~~~~~~~~~~~~~~~~~~~~~~~~~~~~~ 1

               *         *         *         *         *         *         *         *         *         *
 301 aagggttcgaccccagacctgggcagtgaaggtatagctggcagcagtgggcaggtgaggaccgccgtctgctgggcaggtgagtctccttcttctctct 400

   1 ~~~~~~~~~~~~~~~~~~~~~~~~~~~~~~~~~~~~~~~~~~~~~~~~~~~~~~~~~~~~~~~~~~~~~~~~~~~~~~~~~~~~~~~~~~~~~~~~~~~~ 1

               *         *         *         *         *         *         *         *         *         *
 401 tggcctcgctccactgaccttctaaacgaaggtttagagaggggggcctggtgagaagaagcggctggcctcgcagcagaatggcacatagaaaggcagga 500

   1 ~~~~~~~~~~~~~~~~~~~~~~~~~~~~~~~~~~~~~~~~~~~~~~~~~~~~~~~~~~~~~~~~~~~~~~~~~~~~~~~~~~~~~~~~~~~~~~~~~~~~ 1

               *         *         *         *         *         *         *         *         *         *
 501 tagttagcaaaggagacatcgtctcgggggggagccgagacagaaggaggctgggggaccattggcgaccccaggtggaaagagctcttagagagaagaaa 600

   1 ~~~~~~~~~~~~~~~~~~~~~~~~~~~~~~~~~~~~~~~~~~~~~~~~~~~~~~~~~~~~~~~~~~~~~~~~~~~~~~~~~~~~~~~~~~~~~~~~~~~~ 1

               *         *         *         *         *         *         *         *         *         *
 601 gaagaggtgcagggttgccagtaaagactgaggccgctgcctccagggaggtgataggagtccttggagacagtggcagagaccatgggatccagcaaga 700

   1 ~~~~~~~~~~~~~~~~~~~~~~~~~~~~~~~~~~~~~~~~~~~~~~~~~~~~~~~~~~~~~~~~~~~~~~~~~~~~~~~~~~~~~~~~~~~~~~~~~~~~ 1

               *         *         *         *         *         *         *         *         *         *
 701 acagaagcattctaggctggggtcaaacagggcaagatggggtcacaagacacagatgggtccccagccgccacaacatcccacccaccgtaattcactt 800

   1 ~~~~~~~~~~~~~~~~~~~~~~~~~~~~~~~~~~~~~~~~~~~~~~~~~~~~~~~~~~~~~~~~~~~~~~~~~~~~~~~~~~~~~~~~~~~~~~~~~~~~ 1

               *         *         *         *         *         *         *         *         *         *
 801 agaagaaggttcaagagtggctctggcaaagtcccaagtttgccagagcctcaataactggagaatggaaaagaagggcagtgcagggtgtcaccagaag 900

   1 ~~~~~~~~~~~~~~~~~~~~~~~~~~~~~~~~~~~~~~~~~~~~~~~~~~~~~~~~~~~~~~~~~~~~~~~~~~~~~~~~~~~~~~~~~~~~~~~~~~~~ 1

               *         *         *         *         *         *         *         *         *         *
 901 gggagtgggggctgcaggtatcggactccagagggattttacagcaaggaggctgcagtgggtccagcctgcagacacaccattcccatgaggcactgcg 1000

   1 ~~~~~~~~~~~~~~~~~~~~~~~~~~~~~~~~~~~~~~~~~~~~~~~~~~~~~~~~~~~~~~~~~~~~~~~~~~~~~~~~~~~~~~~~~~~~~~~~~~~~ 1

               *         *         *         *         *         *         *         *         *         *
1001 gcccagggactggtgcggaaagggcccacagtggacttggtacactgtatgccctaaccgctcagtccctgggtctggcatgacagacagaacatttcca 1100

   1 ~~~~~~~~~~~~~~~~~~~~~~~~~~~~~~~~~~~~~~~~~~~~~~~~~~~~~~~~~~~~~~~~~~~~~~~~~~~~~~~~~~~~~~~~~~~~~~~~~~~~ 1

               *         *         *         *         *         *         *         *         *         *
1101 ggggagtcaagggcacaggatgaagccagacgaggcgaggcaggcggggcagaatgaatgagtttctagggagggaggttgggtgcaggtagagcgagta 1200

   1 ~~~~~~~~~~~~~~~~~~~~~~~~~~~~~~~~~~~~~~~~~~~~~~~~~~~~~~~~~~~~~~~~~~~~~~~~~~~~~~~~~~~~~~~~~~~~~~~~~~~~ 1

               *         *         *         *         *         *         *         *         *         *
```

```
1201 gctggggtggtgagccagggaggcactggcctccagagtccgtggccaaggagggccttgcgggcggcgacggagcagtgatcggtgtctcgaagagctc 1300

   1 ~~~~~~~~~~~~~~~~~~~~~~~~~~~~~~~~~~~~~~~~~~~~~~~~~~~~~~~~~~~~~~~~~~~~~~~~~~~~~~~~~~~~~~~~~~~~~~~~~~~~ 1

                                                                                                   Exon2
              *         *         *         *         *         *         *         *         *         *
1301 ggactggagactagggtaagtgtctgtcccgctcgtggtcacccagtctcctcccacgcaagttcaattaactcatgtcttcatttctccctatagccag 1400

   1 ~~~~~~~~~~~~~~~~~~~~~~~~~~~~~~~~~~~~~~~~~~~~~~~~~~~~~~~~~~~~~~~~~~~~~~~~~~~~~~~~~~~~~~~~~~~~~~~~~~~~ 1

              *         *         *         *         *         *         *         *         *         *
1401 gtctccagcagaggtggatgtgcctgccagtcactgaaggcgaggatgacaggtgtggtcaatgtgacagaaagacatgacatggtccggtgtgatggag 1500

   1 ~~~~~~~~~~~~~~~~~~~~~~~~~~~~~~~~~~~~~~~~~~~~~~~~~~~~~~~~~~~~~~~~~~~~~~~~~~~~~~~~~~~~~~~~~~~~~~~~~~~~ 1

              *         *         *         *         *         *         *         *         *         *
1501 aggacagaagggcagtcatccagccttcttggtgagcatactccctgccacagggctagtccgctcaaccacctaattgtccacccactcactcaggatt 1600
                                                        |||||||||||||||||||||||||||||||||||||||||||||||||
   1 ~~~~~~~~~~~~~~~~~~~~~~~~~~~~~~~~~~~~~~~~~~~~~~~~~~~AGGGCTAGTCCGCTCAACCACCTAATTGTCCACCCACTCACTCAGGATT 49
                                                                          *         *         *         *

                   Exon3
              *         *         *         *         *         *         *         *         *         *
1601 ctgtcctttgcagaacaccatgggctggcgccttgtcgtagaagccgtctgttctttcacttttcccaaagagctaacacttctctgctgctctctggat 1700
     ||||||||||||||||||||||||||||||||||||||||||||||||||||||||||||||||||||||||||||||||||||||||||||||||||||
  50 CTGTCCTTTGCAGAACACCATGGGCTGGCGCCTTGTCGTAGAAGCCGTCTGTTCTTTCACTTTTCCCAAAGAGCTAACACTTCTCTGCTGCTCTCTGGAT 149
     *         *         *         *         *         *         *         *         *         *

              *         *         *         *         *         *         *         *         *         *
1701 cctcctccccctaccttgaaccctcaagatgaaaggtgagttctcttctgccccatgtgggtgggagagggtgggatgccaaggacaggggtctcattct 1800
     ||||||||||||||||||||||||||||||||||||||||||||||||||||||||||||||||#|||||||||||||||||||||||||||||||||||
 150 CCTCCTCCCCCTACCTTGAACCCTCAAGATGAAAGGTGAGTTCTCTTCTGCCCCATGTGGGTGGAAGAGGGTGGGATGCCAAGGACAGGGGTCTCATTCT 249
     *         *         *         *         *         *         *         *         *         *

                   Exon4
              *         *         *         *         *         *         *         *         *         *
1801 ctcccacccatagaaatggtgctacccagctcatgtctgggcctttgaatccggggacttctttaagtccgtctcgttctgaatcaagaagatgctgcaa 1900
     ||||||||||||||||||||||||||||||||||#|||||||||||||||||||||||||||||||||||||||||||||||||||||||||||||||||
 250 CTCCCACCCATAGAAATGGTGCTACCCAGCTCATATCTGGGCCTTTGAATCCGGGGACTTCTTTAAGTCCGTCTCGTTCTGAATCAAGAAGATGCTGCAA 349
     *         *         *         *         *         *         *         *         *         *

              *         *         *         *         *         *         *         *         *
1901 tcagaaccactacactacctgcctcaggaatctgctccaaggtgagctggggcaccctttggaagcttgccaagcccactccccaccccaccccc-cgcc 1999
     ||||||||||||||||||||||||||||||||||||||||||||||||||||||||||||||||||||||||||||||||||||||||||||||| ||||
 350 TCAGAACCACTACACTACCTGCCTCAGGAATCTGCTCCAAGGTGAGCTGGGGCACCCTTTGGAAGCTTGCCAAGCCCACTCCCCACCCCACCCCCTCGCC 449
     *         *         *         *         *         *         *         *         *         *

                                 Exon5
     *         *         *         *         *         *         *         *         *         *
2000 ccacctcatttgtctttattctctttgcaggtgaagctgaaagaacagatggtgtcaacattttgaaagagcagactcatagcacccacccacccctgag 2099
     ||||||||||||||||||||||||||||||||||||||||||||||||||||||||||||||||||||#|||||||||||||||||||||||||||||||
 450 CCACCTCATTTGTCTTTATTCTCTTTGCAGGTGAAGCTGAAAGAACAGATGGTGTCAACATTTTGAAACAGCAGACTCATAGCACCCACCCACCCCTGAG 549
     *         *         *         *         *         *         *         *         *         *

     *         *         *         *         *         *         *         *         *         *
2100 aatccatcttcatggccaactctgcctgacccgggagaccaccacccacatcatcctggagccaagcctctaccccgggatgacttcatcatctccctcc 2199
     ||||||||||||||||||||||||||||||||||||||||||||||||||||||||||||||||||||||||||||||||||||||||||||||||||||
 550 AATCCATCTTCATGGCCAACTCTGCCTGACCCGGGAGACCACCACCCACATCATCCTGGAGCCAAGCCTCTACCCCGGGATGACTTCATCATCTCCCTCC 649
     *         *         *         *         *         *         *         *         *         *

     *         *         *         *         *         *         *         *         *         *
2200 tgtctttttcttcttcctcctttcctgtaattctgtttctttccttttgttccttccttgcttgagagactcaaagcacccgtgactctgtttccccatt 2299
     ||||||||||||||||||||||||||||||||||||||||||||||||||||||||||||||||||||||||||||||||||||||||||||||||||||
 650 TGTCTTTTTCTTCTTCCTCCTTTCCTGTAATTCTGTTTCTTTCCTTTTGTTCCTTCCTTGCTTGAGAGACTCAAAGCACCCGTGACTCTGTTTCCCCATT 749
     *         *         *         *         *         *         *         *         *         *

     *         *         *         *         *         *         *         *         *         *
2300 taccccctttgaatttgcactaagtcgattgcactggtttggagtcccggagatagctttgagtctctccgtatgaatgtatacagcgagtgtgtaaac 2399
     ||||||||||||||||||||||||||||||||||||||||||||||||||||||||||#|||||||||||||||||||||||||||||#|||||||||#|
 750 TACCCCCTTTGAATTTGCACTAAGTCGATTGCACTGGTTTGGAGTCCCGGAGATAGCCTTGAGTCTCTCCGTATGAATGTATACAGCAAGTGTGTAAGC 849
     *         *         *         *         *         *         *         *         *         *

     *         *         *         *         *         *         *         *         *         *
2400 ctctttggcaatgctgccccagtacccacctgtcgtccatctccgtctgagggcaactgggtgtggccgtgtgcttgaggcctcgccttcccctcgccta 2499
     ||||||||||||||||||||||||||||||||||#||||||||||||||||||||||||||||||||
```

```
 850 CTCTTTGGCAATGCTGCCCCAGTACCCACCTGTCATCCATCTCCGTCTGAGGGCAACTGGGTGTGG~~~~~~~~~~~~~~~~~~~~~~~~~~~~~~~~~~ 915
     *         *         *         *         *         *         *

     *         *         *         *         *         *         *         *         *         *
2500 gtctggaagcagttccatcataaagtgttcaacatgccctacttcatcctttgcccctcctcaccagggcctcaccagaggtcctgggtccatcaataaa 2599

 915 ~~~~~~~~~~~~~~~~~~~~~~~~~~~~~~~~~~~~~~~~~~~~~~~~~~~~~~~~~~~~~~~~~~~~~~~~~~~~~~~~~~~~~~~~~~~~~~~~~~~ 915

     *         *
2600 tacagttacagtcatt 2615

 915 ~~~~~~~~~~~~~~~~ 915
```
