# Supplementary material for: Elevated Expression of H19 and Igf2 in the Female Mouse Eye
Source: PLoS One. 2013 Feb 20;8(2):e56611. doi: 10.1371/journal.pone.0056611 (PMC3577879; doi:10.1371/journal.pone.0056611)
Supplement: Table S1 — Eye H19 intensities from analysis of 1 female and 1 male microarray hybridization in 20 mouse strains/subspecies (1448194_a_at, MOE340 Affymetrix, GeneNetwork depository: http://www.genenetwork.org, Accession number: GN207). RNA pools of 4–8 eyes from 2–4 individuals are included in each hybridization. (PDF) [file pone.0056611.s004.pdf]

Table S1

**Gene Symbol**	**H19**
Probe Set ID	1448194_a_at
Transcript ID(Array Design)	Mm.14802.1
Representative Public ID	NM_023123
Archival UniGene Cluster	Mm.14802
UniGene ID	Mm.14802
Alignments	chr7:149761435-149764027 (-) // 94.2 // q36.1
Ensembl	ENSMUSG00000000031
Entrez Gene	14955
RefSeq Protein ID	---
RefSeq Transcript ID	NR_001592
MGI Name	---

**Strain|Subspecies**	**log2(F)**	**log2(M)**	**F/M_fold-change**
129S1/SVIMJ	9.956	9.508	1.364
BXSB/MpJ	9.526	9.598	0.951
C3H/HEJ	10.056	9.619	1.354
CAST/Ei	10.521	10.023	1.412
CBA/CaJ	10.174	9.750	1.342
CZECHII/EiJ	9.554	8.642	1.882
DBA/2J	10.147	9.875	1.207
FVB/NJ	9.326	9.310	1.011
KK/HIJ	9.351	8.923	1.345
LG/J	9.784	9.479	1.235
LP/J	10.200	10.081	1.086
MOLF/EiJ	9.292	9.192	1.072
NOD/LtJ	9.172	9.029	1.104
NZB/BlNJ	10.087	9.780	1.237
NZO/H1LtJ	9.832	9.519	1.242
NZW/LACJ	9.113	8.810	1.234
PANCEVO/EiJ	9.107	8.606	1.415
PWD/PHJ	8.853	9.206	0.783
PWK/PHJ	8.848	9.111	0.833
WSB/Ei	9.527	9.043	1.399
p(paired two-sided t-test):			0.0006055
Mean F/M_fold-change:			1.225

Strain|Subspecies description

REF|GeneNetwork|http://www.genenetwork.org

129S1/SvImJ : Collaborative Cross strain sequenced by NIEHS; background for many knockouts (R1 ES cell line); Phenome Project A list. This strain (JAX

No 002448, aka 129S1/Sv-++Kitl/+) carries hypopigmentation mutations (white bellied chinchilla) of the tyrosinase gene on Chr 7 and a mutant allele of the steel (Kitl) gene. It is also a *cone photoreceptor function loss 3* mutant (*Cpfl3* allele) of the *Gnat2* gene that is a model for achromatopsia (JAX Stock Number: **002448**)

BXSB/MpJ: A white-bellied agouti strains with interesting autoimmune disease restricted to males that is associated with a mutation in the *Yaa* gene that causes glomerulonephritis, a dramatic increase in number of peripheral monocytes, and pre-B-cell deficiency (JAX Stock Number: **000740**)

C3H/HeJ: The Heston (He) substrain with a wildtype agouti (A allele) coat color. Sequenced by Perlegen/NIEHS; paternal parent of the BXH panel; Phenome Project old group A list. Important to note for this eye expression dataset, C3H/HeJ is a *Pdeb6 rd1* mutant with near total photoreceptor loss at as early as postnatal day 30. Also a *Tlr4* mutant that is endotoxin resistant. (JAX Stock Number: **000659**)

CAST/EiJ: A wild-derived inbred *Mus musculus castaneus* strain. Samples of this subspecies were captured in Southeast Asia. One of three wild-derived strains in the Collaborative Cross sequenced by NIEHS; Phenome Project A list. CAST/Ei and CAST/EiJ are the same strain. The addition of the "J" is trivial and was added when stock were transferred from Dr. Eicher's lab to the Jackson Laboratory production facility in about 2004. (JAX Stock Number:**000928**)

CBA/CaJ: Agouti strain from the Jackson Laboratory. Wildtype pigment genes. (JAX Stock Number: **000654**)

CZECHII/EiJ: Czech 2 is a wild-derived inbred strain *M. musculus musculus* strain. Samples of this subspecies were caught in the Czech Republic and inbred at the Jackson Laboratory by Eva Eicher. White-bellied agouti. (JAX Stock Number: **001144**).

DBA/2J: The dilute, brown, agouti (dba) strain is the oldest inbred strain of mouse. Inbreeding was started in 1909 by Little. A tyrosinase related protein 1 (*Tyrp1 b*) brown allele mutant. A myosin 5a (*Myo5a d*) dilute allele mutant. Sequenced by Perlegen/NIEHS and Celera; paternal parent of the BXD panel; Phenome Project old A group list. (JAX Stock Number: **000671**)

FVB/NJ: Friend's leukemia virus B (FVB) strain. Sequenced by Perlegen/NIEHS and Celera. *Tyr* c locus albino and a *Pdeb6 rd1* mutant derived from Swiss mice at NIH. This has been the most common strain used to make transgenic mice due to large and easily injected oocytes; Phenome Project A list (JAX Stock Number: **001800**).

KK/HlJ: K Kondo's (KK) Kasukabe strain is a homozygous age-related hearing loss (*ahl*) allele mutant of the *Cdh23* gene. A *Tyr* c locus albino strain. Males have a form of type 2 diabetes. Sequenced by Perlegen/NIEHS. (JAX Stock Number: **002106**)

LG/J: Large (LG) strain. Paternal parent of the Large-by-Small set of RI strains made by James Cheverud and colleagues (the LGXSM panel, not to be

confused with the LongXShort or LXS panel). A *Tyr* c locus albino strain. (JAX Stock Number: **000675**)

LP/J: White-bellied agouit strain with a piebald mutation in the endothelin receptor type B *Ednrb* gene from at the Jackson Laboratory. Some reduction in melanocytes in choroid of eye due to neural creast migration abnormalities. (JAX Stock Number: **000676**)

MOLF/EiJ: A wild-derived inbred strain derived from *M. musculus molossinus* samples camputered in Fukuoka, Japan. This strain has the retinal degeneration *rd1* allele in *Pde6b*. There appears to have been some genetic contamination of this strain with conventional inbred strains in the past several decades (F. Pardo, personal communication to RWW, August 2006). However, the strain is currently fully inbred. (JAX Stock Number: **000550**)

NOD/LtJ: Non-obese diabetic strain, originally from M. Hattori in Kyoto, Japan. This is the Edward Leiter (Lt) substrain from the Jackson Laboratory. Collaborative Cross strain sequenced by NIEHS; Phenome Project B list. Homozygous age-related hearing loss (*ahl*) allele mutant of the *Cdh23* gene. A *Tyr* c locus albino strain. (JAX Stock Number: **001976**)

NZO/HlLtJ: New Zealand Obese strain. This is a severely obese and hypertensive strain. Males often develop a type 2 diabetes. Collaborative Cross strain. Agouti coat color. (JAX Stock Number: **002105**)

NZB/BlNJ: New Zealand Black inbred strain from Bielschowsky (BL, substrain is "B lowercase L N", not "BiN") now maintained at the Jackson Laboratory. (JAX Stock Number: **000648**)

NZW/LacJ: New Zealand White strain from the Laboratory Animal Center (Carshalton, UK), now maintained at the Jackson Laboratory. Carries the *Tyr* c locus albino mutation, the pink-eye dilution mutation in the *Oca2* or p locus, and the brown allele at *Tyrp1*. (JAX Stock Number: **001058**)

PANCEVO/EiJ: PANCEVO/EiJ is a wild-derived inbred strain from the *Mus spicilegus* samples caught in the Pancevo, Serbia. This species of mouse is also known as the Steppe mouse (taxon identifier **10103**). *M. spicilegus* is a colonial **mound-building species**. No known ocular or retina mutations, but the expression level of *Gnat2* is low in this strain, either due to a 3' UTR length variant or possible achromatosia (cone degeneration) (JAX Stock Number:**001384**)

PWD/PhJ: A wild-derived *Mus musculus musculus* agouti strain inbred from samples caught near Prague, Czech Republic. Sequenced by Perlegen/NIEHS; parental strain for a consomic set by Forjet and colleagues. (JAX Stock Number: **004660**)

PWK/PhJ: A wild-derived *Mus musculus musculus* inbred strain from samples caught near Lhotka, Czech Republic. Collaborative Cross strain; Phenome Project D list. (JAX Stock Number: **003715**)

WSB/EiJ: Watkin Star line B (or "wild son-of-a-bitch") is a wild-derived *Mus musculus domesticus* inbred strain from samples caught in

Maryland, USA. A Collaborative Cross strain sequenced by NIEHS; Phenome Project C list (JAX Stock Number: **001145**)
